# Clinical evaluation of deep learning and atlas‐based auto‐segmentation for critical organs at risk in radiation therapy

**DOI:** 10.1002/jmrs.618

**Published:** 2022-09-23

**Authors:** Eddie Gibbons, Matthew Hoffmann, Justin Westhuyzen, Andrew Hodgson, Brendan Chick, Andrew Last

**Affiliations:** ^1^ Department of Radiation Oncology Mid North Coast Cancer Institute Port Macquarie New South Wales Australia; ^2^ Department of Radiation Oncology Mid North Coast Cancer Institute Coffs Harbour New South Wales Australia

**Keywords:** artificial intelligence, atlas contouring, auto‐segmentation, deep learning, organs at risk

## Abstract

**Introduction:**

Contouring organs at risk (OARs) is a time‐intensive task that is a critical part of radiation therapy. Atlas‐based automatic segmentation has shown some success at reducing this time burden on practitioners; however, this method often requires significant manual editing to reach a clinically accurate standard. Deep learning (DL) auto‐segmentation has recently emerged as a promising solution. This study compares the accuracy of DL and atlas‐based auto‐segmentation in relation to clinical ‘gold standard’ reference contours.

**Methods:**

Ninety CT datasets (30 head and neck, 30 thoracic, 30 pelvic) were automatically contoured using both atlas and DL segmentation techniques. Sixteen critical OARs were then quantitatively measured for accuracy using the Dice similarity coefficient (DSC) and Hausdorff distance (HD). Qualitative analysis was performed to visually classify the accuracy of each structure into one of four explicitly defined categories. Additionally, the time to edit atlas and DL contours to a clinically acceptable level was recorded for a subset of 9 OARs.

**Results:**

Of the 16 OARs analysed, DL delivered statistically significant improvements over atlas segmentation in 13 OARs measured with DSC, 12 OARs measured with HD, and 12 OARs measured qualitatively. The mean editing time for the subset of DL contours was 50%, 23% and 61% faster (all *P* < 0.05) than that of atlas segmentation for the head and neck, thorax, and pelvis respectively.

**Conclusions:**

Deep learning segmentation comprehensively outperformed atlas‐based contouring for the majority of evaluated OARs. Improvements were observed in geometric accuracy and visual acceptability, while editing time was reduced leading to increased workflow efficiency.

## Graphical abstract

Contouring organs at risk is a time consuming task in radiation therapy. Auto‐segmentation is a tool that can be used to alleviate this time burden on clinicians. Deep learning based auto‐segmentation has been shown to offer improved accuracy and time saving capabilities over atlas‐based techniques.

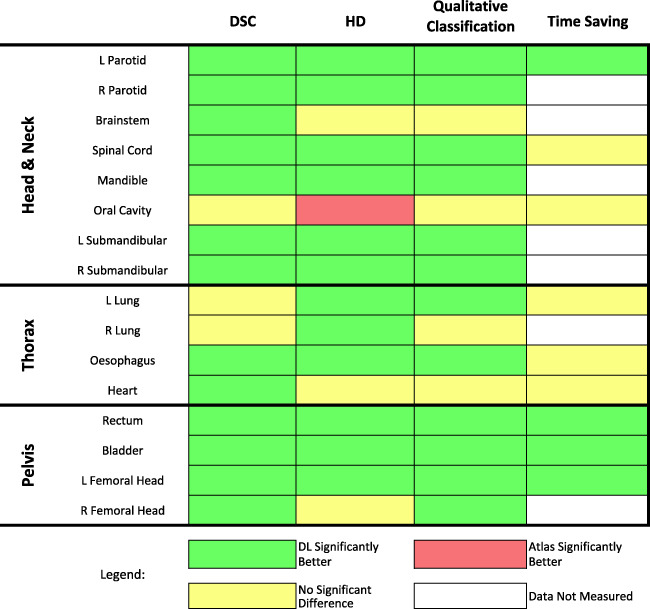

## Introduction

Accurate delineation of organs at risk (OARs) is an important aspect of radiation therapy. Precise contours are required to guide target prescription doses, estimate the likelihood of radiation‐induced side‐effects and optimise dosimetry when inverse planning. Manual segmentation is however a very time and resource‐intensive process, often putting a strain on time‐critical downstream planning tasks. Accurate contouring is also highly dependent on the skill level of the delineator, and OAR consistency can be impacted by inter‐ and intra‐observer variability.^1^, ^2^ Auto‐segmentation aims to reduce the time burden placed on clinicians by using computer algorithms to estimate the position of OARs on planning scans.^3^ Furthermore, it can help limit inter‐ and intra‐observer variability as contour output is consistent and unaffected by human preference or experience.^4^, ^5^

Over the past decade, atlas‐based auto‐segmentation has been a frequently used technique in radiation therapy practice.^3^, ^4^, ^5^, ^6^ Atlases are typically made up of a library of 10–30 expertly contoured datasets that represent a varied range of patient anatomy for a given treatment area.^7^ Atlas software commonly uses deformable image registration to transform contours from one or multiple datasets within the atlas library onto an incoming scan. Due to the limited number of library cases, a lack of user involvement in the initial rigid registration, poor deformations in areas of uniform density and non‐ideal dataset selection, atlas contours often require significant editing to be clinically accurate.^4^, ^8^ Moreover, it has been shown that performance plateaus as the number of library datasets in an atlas is increased.^7^ While atlas‐based auto‐segmentation has been shown to offer some time‐saving improvements over manual contouring, efficiency benefits are generally limited to non‐mobile or bony OARs.^3^, ^4^, ^5^

The recent evolution of machine learning, in particular deep learning (DL), has emerged as a promising solution to overcome the limitations associated with atlas‐based systems. Deep learning auto‐segmentation can make use of multi‐layered convolution ‘neural’ networks (CNNs). CNNs are exposed to large numbers of pre‐contoured datasets and optimised (or ‘trained’) to identify complex non‐linear spatial relationships within those images.^9^, ^10^ This allows DL models to predict structure location on unfamiliar images based on previously studied training data. Pleasingly, multiple groups have reported encouraging results for DL segmentation when compared to atlas‐based techniques.^10^, ^11^, ^12^, ^13^

In this study, we examine the impact of auto‐segmentation on a clinical radiation therapy workflow, with a focus on quantitative, qualitative and time‐saving metrics. As there are significant overheads associated with developing individual DL models, a commercially available DL segmentation programme was compared to locally created atlases. This validated the performance and feasibility of applying externally trained DL models on independent data and provided an assessment against clinically used atlas technology.

## Methods

### Patient selection

In reverse chronological order from 31 December 2020, the computed tomography (CT) images of patients treated curatively with head and neck (H&N), lung, and prostate malignancies at the Mid North Coast Cancer Institute (MNCCI) were assessed for eligibility. The Mid North Coast Human Research Ethics Committee approved the study prior to commencement. Ninety patients were selected to be included in this study (30 H&N, 30 thoracic, 30 pelvic). *Eligibility criteria*: H&N patients did not have significant tumours infiltrating the salivary glands or oral cavity; thorax patients had a gross tumour volume <150 cc confined to either lung; pelvis patients were from the male prostate cohort and did not have a rectal gel spacer or hip prosthesis. The eligibility requirements were chosen to mimic datasets seen during routine clinical practice and to limit excessive abnormalities within OARs. All datasets used to create or train auto‐segmentation models were excluded.

Three‐dimensional planning CT scans were acquired using a Siemens SOMATOM Confidence CT scanner (Siemens, Munich, Germany) with the following median parameters: 120 kVp, 280 mAs_eff_, 2 mm slice thickness, 1.27 mm in‐plane pixel spacing. Contrast‐enhanced scans were performed on 17% of H&N patients, while thoracic and pelvic CTs were captured without contrast. An iterative metal artefact reduction (iMAR) algorithm was used to remove dental artefact when deemed clinically appropriate.

### Reference contours

All ‘gold standard’ reference contours were manually delineated on planning CT datasets to meet institutional contouring protocols. To reduce inter‐observer variability, clinically treated reference contours from three expert radiation oncologists (ROs) were used, who each specialised in one of the three anatomical sites.

Sixteen OARs that were subjectively considered to be the most critical and frequently delineated were selected for evaluation:

- *Head and neck*: parotids (left + right), brainstem, spinal cord, mandible, oral cavity, submandibular glands (left + right).
- *Thorax*: lungs (left + right), heart, oesophagus.
- *Pelvis*: rectum, bladder, femoral heads (left + right).

### Atlas‐based contours

Atlas‐based OARs were generated using Mirada's Embrace*CT* auto‐segmentation software (Mirada Medical Ltd., Oxford, UK). This multi‐atlas approach creates consensus structures from a representative set of pre‐defined library datasets and deforms contours by majority‐vote onto the incoming image using the Lucas‐Kanade Optic Flow algorithm.^8^

Three locally created atlases were evaluated, each containing a library of 15 CT datasets that were curated to meet department‐specific contouring protocols. The performance of each atlas was pre‐validated and established as clinically acceptable within the local setting. To maximise efficiency, Workflow Box 2.4 (Mirada Medical Ltd., Oxford, UK) was used to perform auto‐segmentation as a background task and export the data directly to the treatment planning system (TPS).

### Deep learning contours

A commercially available DL‐based auto‐segmentation software, DLC*Expert* (Mirada Medical Ltd., Oxford, UK), was utilised to generate contours for each anatomical area. The deep CNNs are based on optimised architecture tailored to process imaging data with millions of degrees of freedom.^12^ A 14‐layer 2D multi‐class network initially predicts OAR location at a coarse resolution; this output is then fed into an independently trained 10‐layer organ‐specific network that performs binary classification for each contour. Further technical details of this DL approach have been published by Yang et al.^13^

Three DL models were provided by the vendor and trained on CT datasets external to this study, all of which were curated to meet international or institutional contouring consensus guidelines.^14^, ^15^, ^16^, ^17^ Table 1 provides details on the originating institutions and the total number of datasets used to train each model. As with atlas segmentation, DL contour generation was automated with Workflow Box 2.4.

**Table 1:** Overview of atlas and deep learning model characteristics.

	Head & Neck		Thorax		Pelvis (male anatomy)	
	Atlas	Deep learning	Atlas	Deep learning	Atlas	Deep learning
No. of training datasets	15	698	15	572	15	437
Data type	Non‐contrast CT	Contrast & non‐contrast CT	Non‐contrast CT	Non‐contrast CT	Non‐contrast CT	Non‐contrast CT
Pixel spacing (mm)	1.27	0.938	1.27	0.98	1.27	0.98
Data origin	MNCCI	UMCG	MNCCI	MAASTRO	MNCCI	MAASTRO

Abbreviations: MAASTRO, Maastricht Radiation Oncology, Netherlands; MNCCI, Mid North Cancer Institute, Australia; UMCG, University Medical Centre Groningen, Netherlands.

### Quantitative evaluation

To evaluate the accuracy of atlas and DL‐based structures in relation to reference OARs, contour analysis was performed using the non‐commercial research application Golden Rule (Canis Lupus LLC, Wisconsin, USA). The following quantitative metrics were measured:

#### Dice similarity coefficient (DSC)

Describes the relative spatial overlap between two volumetric contours.^18^ The DSC index ranges from 0 to 1, where a value of 0 indicates no contour overlap, and a value of 1 indicates perfect volumetric overlap. DSC is calculated for two contoured areas *A* and *B* by the formula:

$$\mathrm{DSC}\;\left(A,B\right)=\frac{2\left(A\cap B\right)}{A+B}$$

#### Hausdorff distance (HD)

Describes the maximum Euclidean distance between the closest outer surface points for two contours.^18^ An optimal HD of 0 mm indicates perfect contour surface correlation; larger HDs indicate increasing contour surface errors. The HD between two finite point sets *A* and *B* is defined by:

$$\mathrm{HD}\;\left(A,B\right)=\max\left(h\left(A,B\right),h\left(B,A\right)\right),$$

where *h*(*A*,*B*) is the directed HD and is given by:

hA,B=maxmina−ba∈A,b∈B

### Qualitative evaluation

Atlas‐ and DL‐generated contours were qualitatively assessed for accuracy and classified into one of four explicitly defined categories as shown in Table 2. Auto‐segmentation classifications of 1 or 2 were considered acceptable for clinical use in their unedited form, while classifications of 3 or 4 would be rejected for clinical use. Five expert observers (radiation therapists with 5+ years' contouring experience) were assigned an individual subset of OARs to classify while blinded to the software origin of each contour. To refresh their understanding of each OAR, observers were offered advanced contour training using the software application ProKnow (Elekta, Stockholm, Sweden) prior to the study. Qualitative assessment was performed on all auto‐segmentations for each of the 90 patient datasets.

**Table 2:** Qualitative accuracy classification for automatically generated contours.

Classification	Descriptor	Definition
1	Accept for clinical use; contour is very precise	<3% of axial slices require manual correction
2	Accept for clinical use; minor edits are required	3–10% of axial slices require manual correction
3	Reject for clinical use; moderate edits are required	10–40% of axial slices require manual correction
4	Reject for clinical use; major edits are required	>40% of axial slices require manual correction

### Time evaluation

The same five expert observers were blindly presented with atlas and DL contours and instructed to make any corrections they considered clinically meaningful. A subset of 9 critical OARs (left parotid, spinal cord, oral cavity, left lung, oesophagus, heart, rectum, bladder and left femoral head) were selected for evaluation and measured on 30 datasets randomly selected from the study (10 H&N, 10 thoracic, 10 pelvic). For bi‐lateral OARs, the left side was chosen to maintain consistency. Contour editing time was measured per‐structure and self‐recorded by the observers in a distraction‐free environment. For each OAR and segmentation method, the time result was averaged from all five observers to limit individual contouring bias. Additionally, the time to delineate each OAR manually was measured to provide a baseline time result. Observers were also free to use all contouring tools available in the TPS, and auto‐segmentations could be cleared and completed manually if the observer predicted this would be more time efficient than adjusting each incorrect slice. The time to make this determination and review each structure was included. Initial auto‐segmentation generation time was not considered in the measurement as this process is automated and does not require additional clinical resources.

### Statistical analysis

For the quantitative and qualitative analysis, a Wilcoxon signed rank test was performed to check for statistical significance. For the time analysis, RM‐ANOVA was used to compare the three segmentation methods (manual, atlas and DL), followed by individual comparisons with Bonferroni corrections. *P*‐values <0.05 (two‐tailed) were considered to be statistically significant. MedCalc v19.04 (MedCalc Software, Ostend, Belgium) was used for statistical comparisons.

## Results

### Quantitative analysis

*For H&N OARs*, statistically significant median DSC improvements were observed in the DL‐based parotids (0.80_atlas_/0.87_DL_), brainstem (0.83_atlas_/0.85_DL_), spinal cord (0.82_atlas_/0.86_DL_), mandible (0.91_atlas_/0.94_DL_) and submandibular glands (0.68_atlas_/0.80_DL_). Median HD reductions were significant for the DL‐based parotids (18.5 mm_atlas_/10.7 mm_DL_), spinal cord (5.2 mm_atlas_/2.9 mm_DL_), mandible (15.8 mm_atlas_/12.2 mm_DL_) and submandibular glands (8.8 mm_atlas_/6.9 mm_DL_). The HD of the atlas‐based oral cavity outperformed DL (11.6 mm_atlas_/12.8 mm_DL_). There were no notable differences between H&N datasets scanned with or without iMAR or contrast enhancement.

*For thoracic OARs*, atlas and DL DICE results were comparable (*P* > 0.05) for the lungs (0.98_atlas & DL_) and heart (0.96_atlas & DL_). Atlas segmentation of the oesophagus showed poor DSC (0.48) correlation with the reference contour, but this increased considerably with DL (0.74). Superior HD outcomes were measured in the DL‐based lungs (22.4 mm_atlas_/18.2 mm_DL_) and oesophagus (18.8 mm_atlas_/12.4 mm_DL_).

*For pelvic OARs*, DSC analysis indicated that DL outperformed atlas segmentation for all four structures: rectum (0.77_atlas_/0.87_DL_), bladder (0.88_atlas_/0.96_DL_), left and right femoral heads (0.96_atlas_/0.98_DL_). Significant HD improvements were observed in the rectum (12.3 mm_atlas_/9.6 mm_DL_), bladder (19.3 mm_atlas_/12.8 mm_DL_) and left femoral head (9.0 mm_atlas_/6.8 mm_DL_).

**Figure 1:**
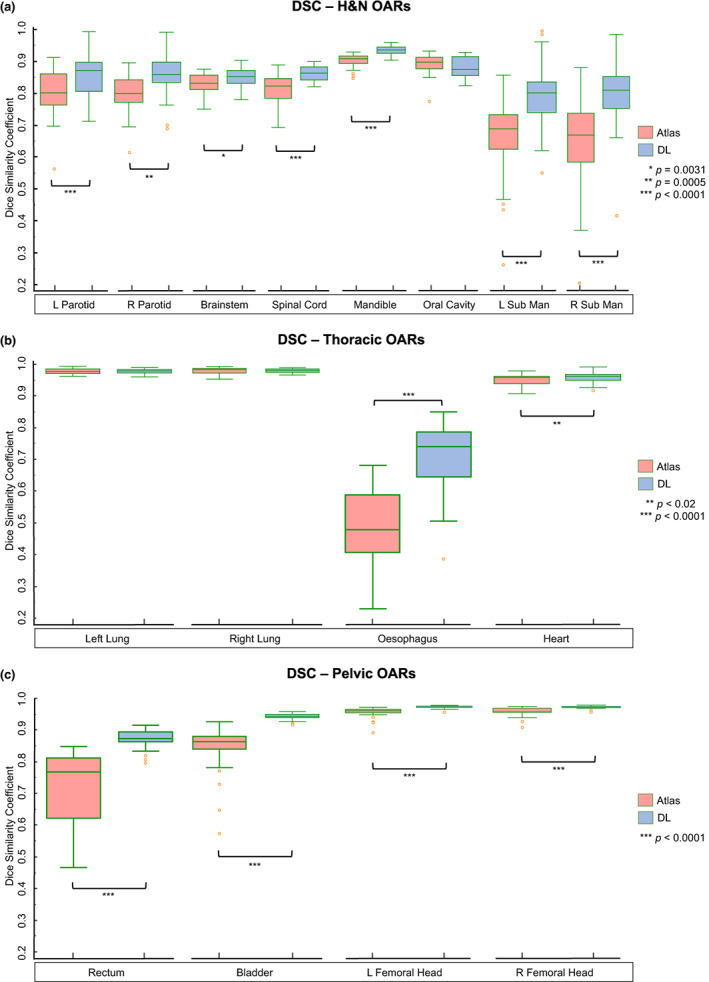
Boxplots showing the Dice similarity coefficient (DSC) of atlas (red) and deep learning (DL) (blue) segmentations when compared against reference contours. Significant differences (*P* < 0.05, ranked Wilcoxon test) between atlas and DL results are indicated (*). Sub Man, Submandibular gland.

**Figure 2:**
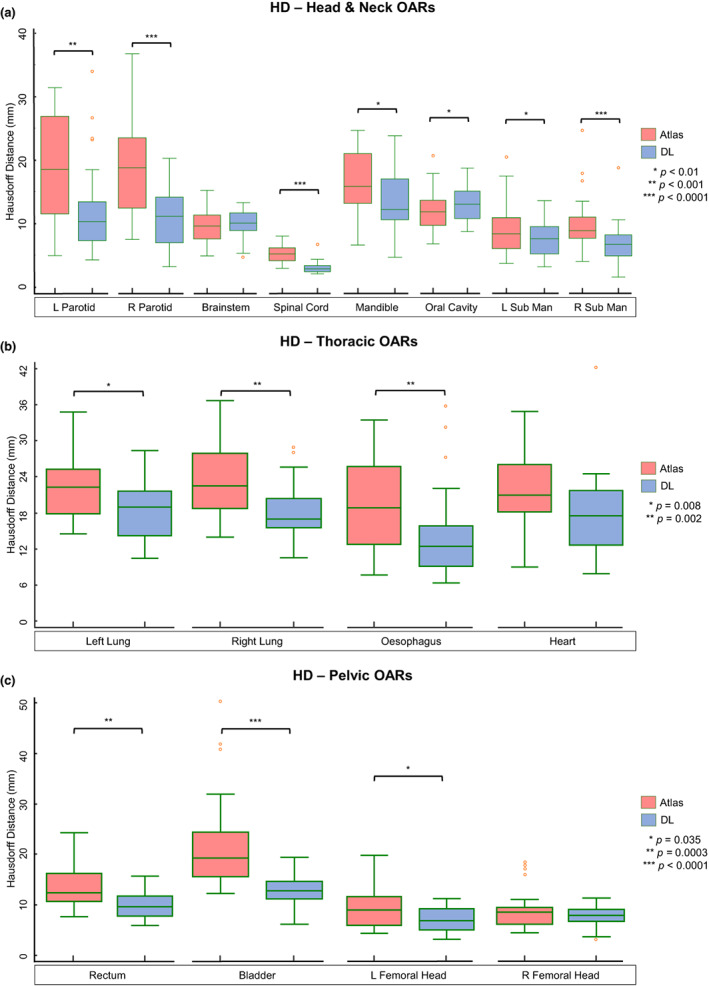
Boxplots showing the Hausdorff distance (HD) of atlas (red) and deep learning (DL) (blue) segmentations when compared against reference contours. Significant differences (*P* < 0.05, ranked Wilcoxon test) between atlas and DL results are indicated (*). Sub Man, submandibular gland.

Boxplots showing all quantitative results are given in Figures 1 and 2. Further analytical results including *P*‐values for each structure are listed in Table S1.

### Qualitative analysis

Deep learning H&N OARs were classified as clinically acceptable (a score of 1 or 2) in 86.3% (55.8%_atlas_) of cases, indicating the majority of these structures were perceived to be suitable for use with only minor or no manual adjustment required. The parotids and submandibular glands experienced considerable accuracy enhancements, with the median classification improving from 3_atlas_ to 2_DL_ (*P* < 0.0001). Spinal cord segmentation performance was excellent, with 14_atlas_ and 25_DL_ structures out of 30 assigned a perfect score of 1.

Clinically acceptable contours were subjectively observed in 69.2% of DL‐based thoracic OARs compared with 54.2% for atlas structures. The left lung was regularly classified as ideal when contoured with DL, a significant improvement over the atlas (2_atlas_/1_DL_). Both auto‐segmentation methods struggled to generate initial oesophagus contours that were deemed acceptable for clinical use (0%_atlas_/13.3%_DL_).

Of the three anatomical sites, OARs in the pelvis experienced the most dramatic increase in perceived accuracy and clinical acceptability (28.3%_atlas_/81.7%_DL_). Significant classification improvements were seen in the bladder (4_atlas_/2_DL_), rectum (4_atlas_/2_DL_) and femoral heads (2_atlas_/1_DL_). Atlas segmentation was unable to produce any bladder or rectum contours that were classified as clinically accurate; conversely, DL did so in 86.6% and 53.3% of cases respectively.

**Figure 3:**
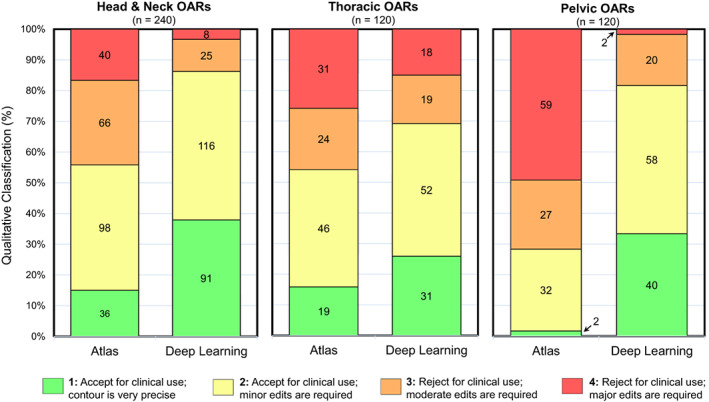
Stacked bar chart of the qualitative four‐grade system used to classify the accuracy of atlas and deep learning‐based contours. The numbers in the bars indicate the observed frequency for each of the four categories. OARs, organs at risk.

Example datasets visually comparing atlas and DL segmentations are shown in Figure S1. Figure 3 shows the grouped qualitative results for each of the 3 anatomical sites; this is further broken down per OAR in Figure S2. *P*‐values are given in Table S2.

### Time analysis

**Figure 4:**
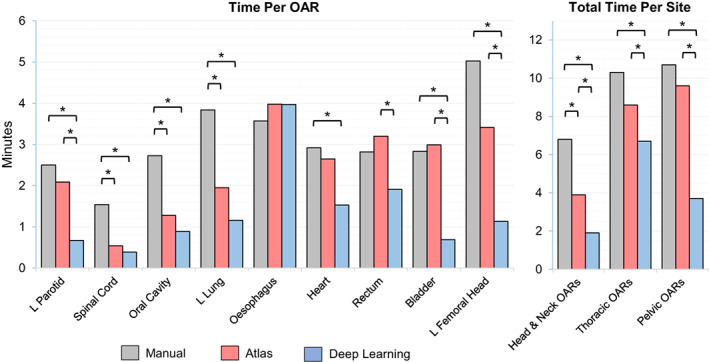
Mean contour adjustment time for manual (grey), atlas (red) and deep learning (blue) segmentation methods. * indicates a significant time difference when comparing two techniques (*P* < 0.05, RM‐ANOVA with individual Bonferroni corrections). OARs, organs at risk.

The mean manual delineation time for the subset of 9 time‐evaluated structures was 6.8 mins, 10.3 mins and 10.7 mins for the H&N, thorax and pelvis respectively. The time required to edit the same atlas‐based contours was 3.9 mins (−42%), 8.6 mins (−17%) and 9.6 mins (−10%); DL‐adjusted structures were completed in 1.9 mins (−71%), 6.7 mins (−36%) and 3.7 mins (−65%). The recorded times for each OAR are shown in Figure 4; *P*‐values can be found in Table S3.

Deep learning segmentation delivered significant time improvements for the left parotid (0.7 min) when compared to both atlas (2.1 min) and manual contouring (2.5 min). Auto‐segmentation of the spinal cord required minimal editing time on average (0.5 min_atlas_/0.4 min_DL_), of which most could likely be attributed to the observer reviewing the acceptability of the contour.

Contour editing time for the atlas and DL‐based left lung (1.9 min_atlas_/1.2 min_DL_) significantly outperformed manual delineation (3.8 min). Curiously, manual delineation (3.6 min) of the oesophagus was slightly more time efficient than atlas (4.0 min) and DL (4.0 min) methods, although this difference was not significant (*P* > 0.05).

The DL‐based rectum was the most time efficient of the 3 contouring techniques (2.8 min_manual_/3.2 min_atlas_/1.9 min_DL_). Bladder performance was comparable for manual (2.8 min) and atlas (3.0 min) methods, but significantly faster with DL (0.7 min). For the left femoral head, DL segmentation (1.1 min) outperformed both atlas (3.4 min) and manual delineation (5.0 min).

## Discussion

**Figure 5:**
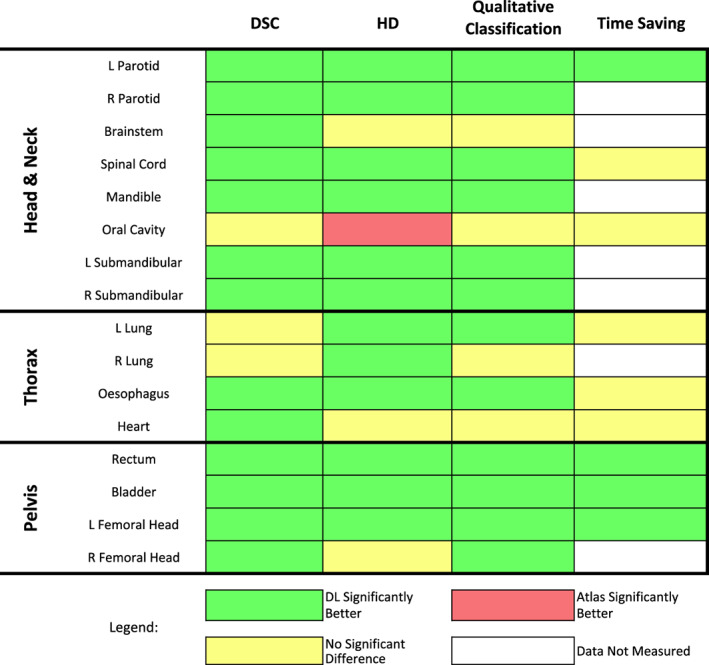
Overview of the quantitative, qualitative and time‐saving results for all organs at risk. Green boxes indicate deep learning (DL) outperformed atlas segmentation with a statistically significant result (*P* < 0.05). Orange boxes indicate atlas segmentation significantly outperformed DL.

In this study, we evaluated multiple DL‐based anatomical models from a single commercial vendor and compared them to current atlas‐based auto‐segmentation practices. By using local data for validation, we were able to assess the performance and feasibility of applying externally trained DL software to an independent radiation therapy workflow. A summary of the results for each auto‐segmentation method is given in Figure 5.

The DL‐based OARs that experienced the most significant quantitative and qualitative accuracy improvements were the rectum, bladder, parotids, submandibular glands and oesophagus. Interestingly, many of these structures are considered to be mobile or elastic organs, meaning they can be susceptible to high levels of anatomic variability between patients. Van Dijk et al. proposed that DL models offer superior segmentation performance over atlas‐based systems due to the vastly increased number of datasets used for training^11^ (our study: *n* = 15_atlas_ vs. ~400‐700_DL_). Additional training data will naturally incorporate a more diverse range of patient anatomy allowing for more robust contours, particularly in regions that experience considerable variability.

It is generally hypothesised that auto‐segmentation accuracy enhancements will likely correlate to time efficiency improvements for clinicians. Lustberg et al. proved this theory by demonstrating that contour editing time was reduced as qualitative accuracy increased for thoracic OARs.^12^ This was further explored by Vaassen et al., who reported a moderate correlation between time savings and DSC and HD outcomes.^19^ The results from our study showed the most significant time‐based OAR improvements (rectum, bladder, left parotid and left femoral head) were also associated with a considerable increase in quantitative and qualitative accuracy. This aligns with the published literature, although it should be noted that there is no perfect metric to predict time‐saving potential. Surface DSC and added path length have recently been presented as promising surrogate metrics;^19^, ^20^ however, a direct measurement of contour adjustment time in a test environment is ideal.^10^, ^11^, ^12^

It is pleasing to see contour accuracy has progressed significantly with DL technology; however, for some OARs, performance output can be sub‐optimal. Qualitative analysis showed that the DL segmented rectum (47%), heart (27%), submandibular glands (32%) and oesophagus (87%) were consistently classified as being unacceptable for clinical use (a score of 3 or 4). Moreover, DL segmentation of the oesophagus often failed to produce a contour on every relevant cross‐sectional slice, leaving it only partially delineated. The aforementioned OARs typically have low contrast boundaries on CT that can be difficult to define for expert human delineators, so it is unrealistic to assume computer‐generated structures will be highly precise. It should also be noted that the qualitative classification scale (Table 2) might be less forgiving to OARs with smaller volumes given their limited number of axial slices.

Curiously, our results highlighted a number of OARs where manual delineation was more time efficient than auto‐segmentation (rectum_atlas_, bladder_atlas_, oesophagus_atlas & DL_). Software‐generated contours with poor geometric accuracy can require editing on every axial slice which is a time‐consuming process. We theorise that the interpolate tool used during manual delineation contributes to this time efficiency outperformance, as only every second or third slice necessitates user input. A potential solution may be to instruct the auto‐segmentation algorithm to apply a contour on every 2nd or 3rd slice for OARs that are commonly inaccurate. Following user review and adjustment, interpolation can be performed. This functionality is available in Workflow Box 2.4, however was not evaluated as part of this work.

It is not always feasible for individual centres to train personalised DL models due to constraints on time, data, resources or staff expertise. Instead, it may be more practical to utilise robust pre‐trained generic models based on accepted contouring consensus guidelines. However, a drawback of this method is that contour output is not optimised to meet individual protocols. Our study showed the HD of the atlas‐based oral cavity significantly outperformed DL (11.6 mm_atlas_/12.8 mm_DL_). This is likely due to differences in how each auto‐segmentation algorithm was programmed to delineate air gaps in relation to our protocol. Transfer learning has been proposed as an approach to independently optimise existing generic models using a small number of new training datasets.^21^ This method requires far fewer resources than building a DL model from scratch and allows segmentation output to be refined to meet individual needs. A study by Brouwer et al. measured the manual adjustments made to DL contours before they were used clinically^22^; future research may look at using this methodology to re‐train and optimise existing DL models to improve performance.

In a busy radiation oncology department, the time efficiencies attributed to DL segmentation can allow staff resources to be redirected towards more complex technical tasks such as plan optimisation, peer review or adaptive workflows. Additionally, shorter treatment planning times may lead to increased patient throughput and reduced wait list pressure. Although not explicitly measured in this study, it is assumed improvements to auto‐segmentation accuracy will help to reduce inter‐observer variability as fewer user adjustments are required. This is particularly beneficial for dose reporting and clinical trials research, where the standardisation of contours is critical.

## Conclusions

Deep learning segmentation has comprehensively outperformed atlas‐based contouring for the majority of OARs in the H&N, thorax and pelvis. Significant qualitative and quantitative accuracy improvements have been observed, and the average editing time required for most structures has been reduced considerably. The application of DL auto‐segmentation will likely lead to efficiency improvements within a radiation therapy workflow. While extensive developments have been made over atlas‐based technology, performance has not yet reached the accuracy level of an experienced clinician, meaning DL structures still require review prior to clinical use.

## Funding Information

None.

## Conflict of Interest

The Mid North Coast Cancer Institute (MNCCI) has a non‐financial research agreement with Mirada Medical Ltd. (Oxford, UK). A time‐limited DLC*Expert* software licence was provided by Mirada Medical Ltd. to undertake this research. MNCCI was supported by Elekta (Stockholm, Sweden) in provision of a time‐limited ProKnow software licence used for this project.

## Ethics Approval

This study was approved as a Quality Improvement Project by the Mid North Coast Human Research Ethics Committee in accordance with section 5.1.22 of the National Statement on Ethical Conduct in Human Research (2007). This decision was reached on the basis that the project is a negligible risk activity analysing already collected de‐identified health information.

## Patient Consent

Informed consent was obtained prior to patient datasets being included in the study.

## Supporting information


**Figure S1.** Example datasets showing manual reference contours (green), atlas contours (red), and deep learning contours (blue) for the head & neck (A), thorax (B), and pelvis (C).
**Figure S2.** Stacked bar chart of the qualitative four‐grade system used to classify the accuracy of atlas and deep learning‐based contours.
**Table S1.** Quantitative results table for all measured organs at risk (OARs).
**Table S2.** Qualitative classification results table for all measured organs at risk (OARs).
**Table S3.** Time analysis results table for the subset of measured organs at risk.

## Data Availability

Research data are stored in an institutional repository and will be shared upon request with the corresponding author.
