# Supplementary material for: Clinical evaluation of deep learning and atlas‐based auto‐segmentation for critical organs at risk in radiation therapy
Source: J Med Radiat Sci. 2022 Sep 23;70(Suppl 2):15–25. doi: 10.1002/jmrs.618 (PMC10122925; doi:10.1002/jmrs.618)
Supplement: Supplementary file 1 — Figure S1. Example datasets showing manual reference contours (green), atlas contours (red), and deep learning contours (blue) for the head & neck (A), thorax (B), and pelvis (C). Figure S2. Stacked bar chart of the qualitative four‐grade system used to classify the accuracy of atlas and deep learning‐based contours. Table S1. Quantitative results table for all measured organs at risk (OARs). Table S2. Qualitative classification results table for all measured organs at risk (OARs). Table S3. Time analysis results table for the subset of measured organs at risk. [file JMRS-70-15-s001.docx]

**Supporting Information**

**Clinical evaluation of deep learning and atlas-based auto-segmentation for critical organs at risk in radiation therapy**

**Journal of Medical Radiation Sciences**

**Authors:**

Eddie Gibbons, B.Sc.(RT).,^a^ Matthew Hoffmann, B.I.T.,^a^ Justin Westhuyzen, M.Sc. Ph.D.,^b^ Andrew Hodgson, B.Sc.(RT).,^a^ Brendan Chick, Ph.D.,^a^ Andrew Last, D.Phil. FRCR.^a^

**Affiliation:**

^a^ Department of Radiation Oncology, Mid North Coast Cancer Institute, Port Macquarie, New South Wales, Australia

^b^ Department of Radiation Oncology, Mid North Coast Cancer Institute, Coffs Harbour, New South Wales, Australia

**Corresponding Author:**

Eddie Gibbons

Email: [eddie.gibbons@health.nsw.gov.au](mailto:eddie.gibbons@health.nsw.gov.au), [eddie.gibbons@uon.edu.au](mailto:eddie.gibbons@uon.edu.au)


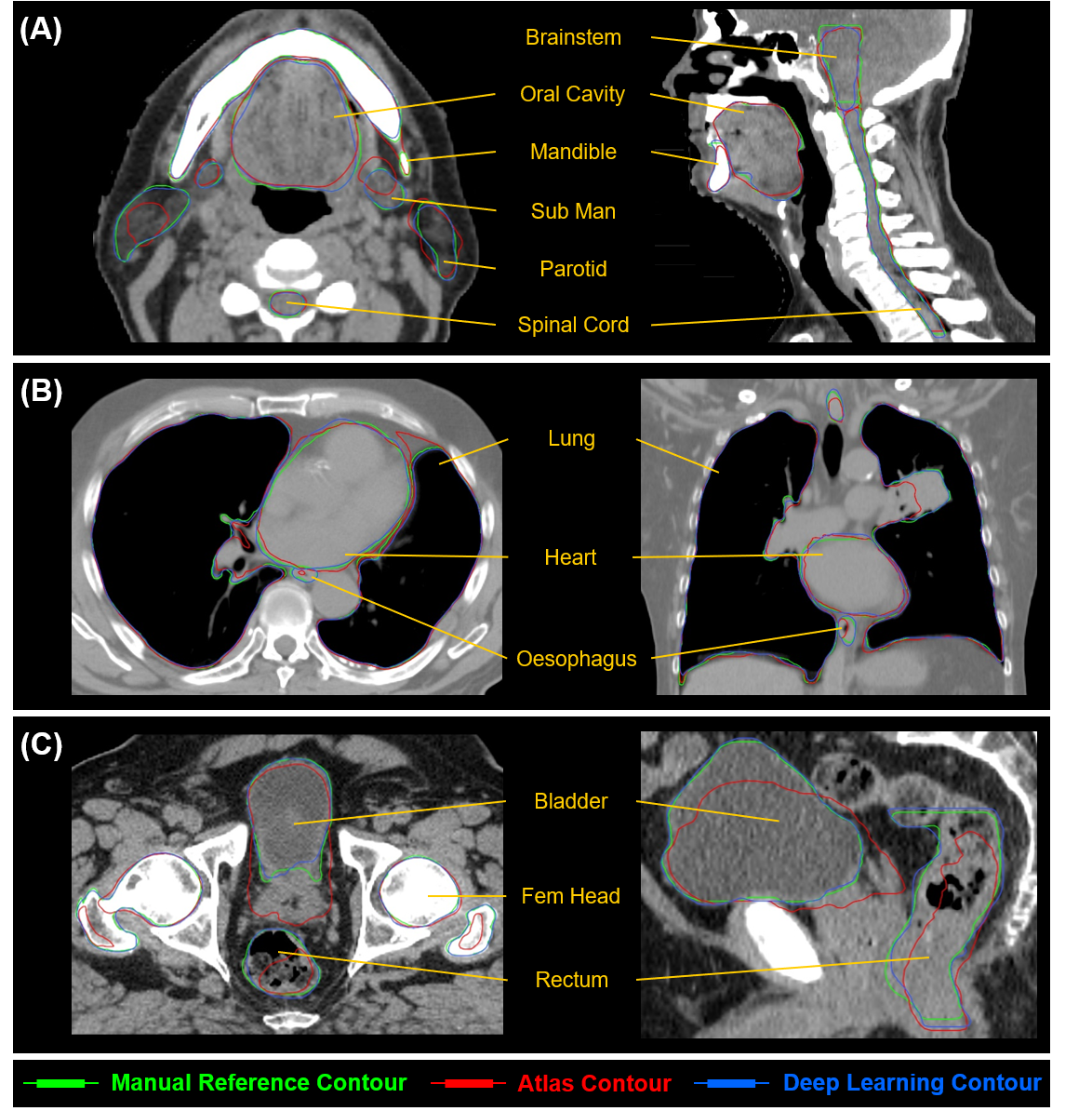
**Supporting Figure 1:** Example datasets showing manual reference contours (green), atlas contours (red), and deep learning contours (blue) for the head & neck (A), thorax (B), and pelvis (C).

*Abbreviations*: Sub Man = Submandibular gland; Fem Head = Femoral head.


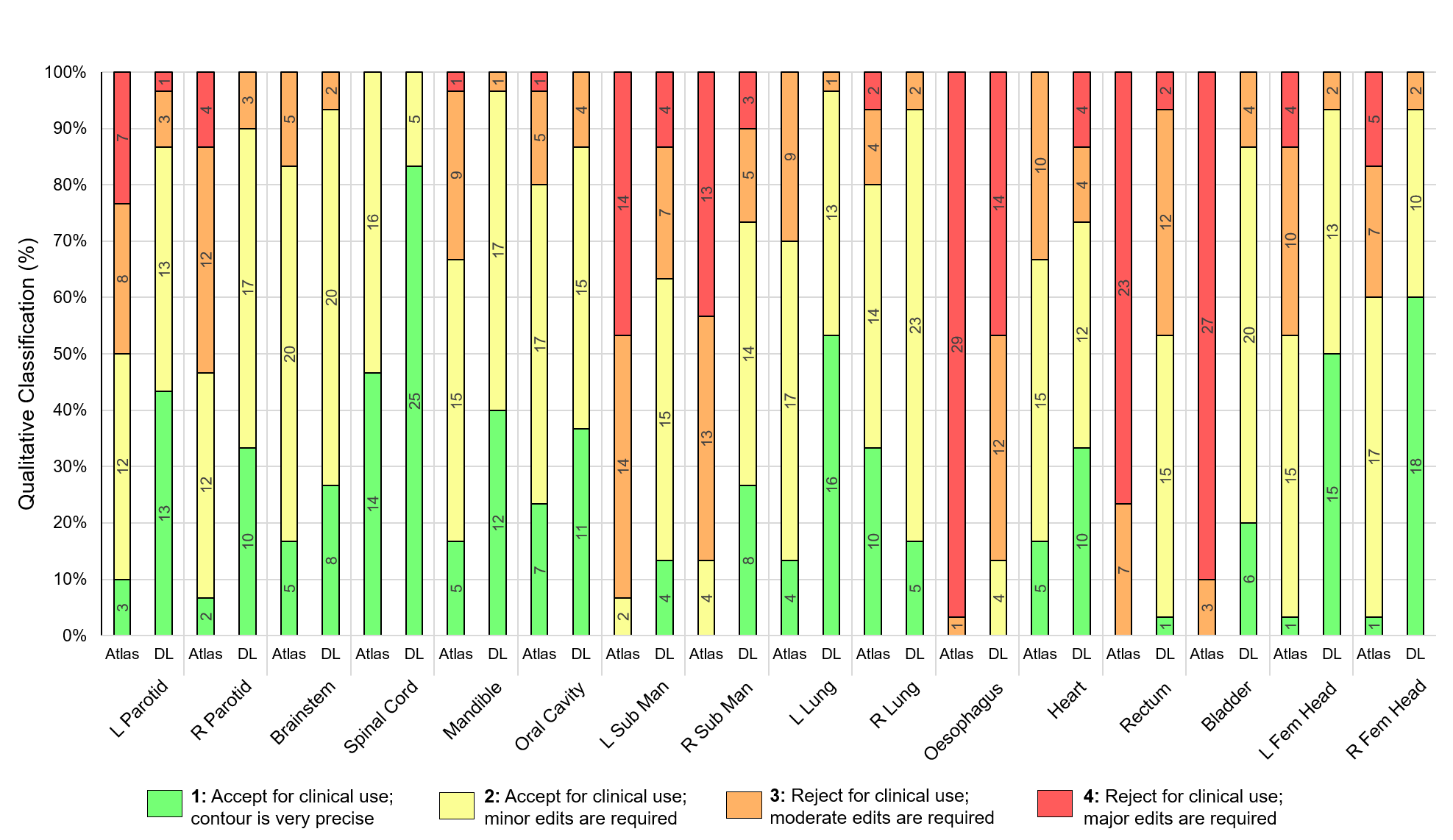
**Supporting Figure 2:** Stacked bar chart of the qualitative four-grade system used to classify the accuracy of atlas and deep learning-based contours. For each organ at risk, 30 classifications were made by the expert observers. The numbers in the bars indicate the frequency for each of the four categories.

*Abbreviations*: DL = Deep learning; Sub Man = Submandibular gland; Fem Head = Femoral head.

**Supporting Table 1:** Quantitative results table for all measured organs at risk (OARs). Red *p*-values indicate OARs that did not reach significance (*p*<0.05, ranked Wilcoxon test). * indicates atlas outperformed deep learning with a significant result.

|  | **DSC** | | | | | **Hausdorff Distance (mm)** | | | | |
| --- | --- | --- | --- | --- | --- | --- | --- | --- | --- | --- |
|  | **Atlas** | | **Deep Learning** | | ***p*-value** | **Atlas** | | **Deep Learning** | | ***p*-value** |
|  | **Median** | **IQR** | **Median** | **IQR** |  | **Median** | **IQR** | **Median** | **IQR** |  |
| L Parotid | 0.801 | 0.77-0.86 | 0.873 | 0.81-0.90 | 0.0001 | 18.38 | 11.5-26.7 | 10.33 | 7.4-13.4 | 0.0006 |
| R Parotid | 0.798 | 0.77-0.84 | 0.858 | 0.83-0.89 | 0.0005 | 18.68 | 12.5-23.3 | 11.14 | 7.0-14.1 | 0.0001 |
| Brainstem | 0.832 | 0.81-0.86 | 0.853 | 0.83-0.87 | 0.0031 | 9.60 | 7.7-11.2 | 10.04 | 8.9-11.6 | 0.6987 |
| Spinal Cord | 0.823 | 0.79-0.85 | 0.863 | 0.84-0.88 | 0.0001 | 5.20 | 4.2-6.2 | 2.89 | 2.5-3.3 | <0.0001 |
| Mandible | 0.908 | 0.90-0.92 | 0.936 | 0.93-0.94 | <0.0001 | 15.80 | 13.2-20.6 | 12.18 | 10.6-16.7 | 0.0029 |
| Oral Cavity | 0.890 | 0.87-0.91 | 0.866 | 0.85-0.91 | 0.0641 | 11.63 | 9.5-13.4 | 12.80 | 10.7-14.8 | *0.0052 |
| L Submandibular | 0.688 | 0.63-0.73 | 0.802 | 0.74-0.84 | <0.0001 | 8.36 | 6.1-10.7 | 7.58 | 5.2-9.5 | 0.0104 |
| R Submandibular | 0.669 | 0.59-0.74 | 0.811 | 0.76-0.85 | <0.0001 | 8.81 | 7.7-11.0 | 6.72 | 4.9-8.2 | 0.0001 |
| L Lung | 0.977 | 0.97-0.99 | 0.979 | 0.97-0.98 | 0.230 | 22.31 | 17.8-25.1 | 18.90 | 14.4-21.5 | 0.0078 |
| R Lung | 0.983 | 0.97-0.99 | 0.981 | 0.98-0.99 | 0.660 | 22.47 | 19.0-27.9 | 16.99 | 15.6-20.4 | 0.0021 |
| Oesophagus | 0.479 | 0.41-0.59 | 0.740 | 0.65-0.79 | <0.00001 | 18.83 | 13.5-25.4 | 12.39 | 9.3-15.8 | 0.0016 |
| Heart | 0.959 | 0.94-0.96 | 0.963 | 0.95-0.97 | 0.0203 | 21.00 | 18.3-25.3 | 17.56 | 12.9-21.6 | 0.1211 |
| Rectum | 0.769 | 0.63-0.81 | 0.873 | 0.86-0.90 | <0.0001 | 12.34 | 10.7-16.0 | 9.63 | 7.8-11.6 | 0.0003 |
| Bladder | 0.879 | 0.86-0.90 | 0.962 | 0.96-0.97 | <0.00001 | 19.27 | 15.8-23.9 | 12.84 | 11.3-14.7 | <0.00001 |
| L Femoral Head | 0.967 | 0.96-0.97 | 0.978 | 0.97-0.98 | <0.00001 | 8.99 | 6.0-11.5 | 6.83 | 5.0-9.1 | 0.0349 |
| R Femoral Head | 0.963 | 0.96-0.97 | 0.977 | 0.97-0.98 | <0.00001 | 8.52 | 6.3-9.5 | 7.96 | 6.7-9.0 | 0.2757 |

*Abbreviations*: IQR = Interquartile range.

|  | **Qualitative Classification** | | | | |
| --- | --- | --- | --- | --- | --- |
|  | **Atlas** | | **Deep Learning** | | ***p*-value** |
|  | **Median** | **Range** | **Median** | **Range** |  |
| L Parotid | 2.5 | 1-4 | 2 | 1-4 | <0.0001 |
| R Parotid | 3 | 1-4 | 2 | 1-3 | 0.0002 |
| Brainstem | 2 | 1-3 | 2 | 1-3 | 0.2078 |
| Spinal Cord | 2 | 1-2 | 1 | 1-2 | 0.001 |
| Mandible | 2 | 1-4 | 2 | 1-3 | 0.0006 |
| Oral Cavity | 2 | 1-4 | 2 | 1-3 | 0.0942 |
| L Submandibular | 3 | 2-4 | 2 | 1-4 | <0.0001 |
| R Submandibular | 3 | 2-4 | 2 | 1-4 | <0.0001 |
| L Lung | 2 | 1-3 | 1 | 1-3 | 0.0007 |
| R Lung | 2 | 1-4 | 2 | 1-3 | 0.8408 |
| Oesophagus | 4 | 3-4 | 3 | 2-4 | 0.0001 |
| Heart | 2 | 1-3 | 2 | 1-4 | 0.5949 |
| Rectum | 4 | 3-4 | 2 | 1-4 | <0.00001 |
| Bladder | 4 | 3-4 | 2 | 1-3 | <0.00001 |
| L Femoral Head | 2 | 1-4 | 1.5 | 1-3 | <0.00001 |
| R Femoral Head | 2 | 1-4 | 1 | 1-3 | <0.00001 |

**Supporting Table 2:** Qualitative classification results table for all measured organs at risk (OARs). Red *p*-values indicate OARs that did not reach significance (*p*<0.05, ranked Wilcoxon test).

**Supporting Table 3:** Time analysis results table for the subset of measured organs at risk. Significant *p*-values are listed for paired segmentation techniques (*p*<0.05, RM-ANOVA comparison with individual Bonferroni corrections). * indicates manual delineation outperformed deep learning with a significant result.

|  | **Time Analysis (min)** | | | | | | | | |
| --- | --- | --- | --- | --- | --- | --- | --- | --- | --- |
|  | **Manual** | | **Atlas** | | **Deep Learning** | | ***p*-value (signif.)** | | |
|  | **Mean** | **SD** | **Mean** | **SD** | **Mean** | **SD** | **Manual v Atlas** | **Atlas**  **v DL** | **Manual v DL** |
| L Parotid | 2.50 | ±0.54 | 2.09 | ±0.68 | 0.69 | ±0.48 |  | <0.0001 | <0.0001 |
| Spinal Cord | 1.54 | ±0.40 | 0.54 | ±0.20 | 0.39 | ±0.19 | 0.0002 |  | <0.0001 |
| Oral Cavity | 2.73 | ±0.55 | 1.28 | ±0.58 | 0.89 | ±0.32 | <0.0001 |  | <0.0001 |
| L Lung | 3.84 | ±1.71 | 1.95 | ±1.06 | 1.16 | ±0.91 | 0.0026 |  | 0.002 |
| Oesophagus | 3.57 | ±0.79 | 3.98 | ±0.77 | 3.97 | ±0.77 |  |  | *0.0458 |
| Heart | 2.92 | ±0.63 | 2.65 | ±1.07 | 1.53 | ±0.64 |  |  | 0.0006 |
| Rectum | 2.82 | ±0.84 | 3.20 | ±0.74 | 1.91 | ±0.62 |  | 0.0042 |  |
| Bladder | 2.83 | ±0.54 | 2.99 | ±1.02 | 0.69 | ±0.45 |  | <0.0001 | <0.0001 |
| L Femoral Head | 5.03 | ±1.56 | 3.41 | ±1.32 | 1.13 | ±0.74 |  | 0.0033 | 0.0007 |

*Abbreviations*: DL = Deep learning; SD = Standard deviation.
